# The Use of Tobacco by Boys

**Published:** 1884-03

**Authors:** 


### The Use of Tobacco by Boys.

In the course of an article on this subject, Dr. Edward O. Otis (*Boston M. and S. Journal*, January 31, 1884,) says:

The evil effects, then, of tobacco upon boys which can be said to have been fairly proved by observation, are—

1. An interference with and impairment of the general development, physically and mentally. Probably it does this by retarding progressive cell change, and impairing nutrition. In an adult, the development is attained, consequently he can smoke with impunity so far as this effect is concerned, when the boy only does it with injury.

2. The tobacco heart. This likewise happens to the adult, but is, I should think, more likely to happen to the youth, and with graver result, because his is such an unstable age physically. For this same reason, the other injurious effects are more liable to be intensified in the youth.

3. Defective muscular co-ordination. I have but little proof to show that this condition is more than temporary, continuing only while the tobacco is used.

4. It reduces the intellectual power of the boy. It does this either by opposing mental application and effort, or else by producing deterioration of the intellect, probably both to a greater or less extent.

5. It impairs the memory. What proof I have adduced seems to show that this is a permanent injury.

6. It may cause defective vision, and also irritation, more or less chronic, to the external parts of the eye.

7. It may unduly excite the sexual system, and lead on to excesses. I have no positive proof of this, but my observation among boys rather points to it.

8. Irritation of the mucous membranes of the mouth and throat, more especially in case of cigarette smoking.

9. It may impair digestion, and produce other derangements, such as habitual uneasiness, hypochondriasis, and so on.

---

Rendering Rubber Gas Tubing Odorless.—The Mohrisch-Schleisher *Gewerbhalle* recommends for deodorizing rubber gas tubing, that equal volumes of 36 per cent. alcohol and good linseed oil be shaken together to form a homogeneous mixture, and the moderately-stretched tube rubbed with a small rag, on which has been dropped a little of the mixture, until quite dry. The operation is to be repeated three or four times at intervals of a few days. The flexibility or color of the tube is said not to be impaired, while the rubber is rendered gas-tight.

---

Important lesson to all writers, as given by Dr. Billings, U. S. A.: 1. Have something to say. 2. Say it. 3. Stop when you have said it. 4. Give a proper title to what you have said.

